# Pseudo-bistability of viscoelastic shells

**DOI:** 10.1098/rsta.2022.0026

**Published:** 2023-04-03

**Authors:** Yuzhen Chen, Tianzhen Liu, Lihua Jin

**Affiliations:** ^1^ Department of Aeronautics and Astronautics, Fudan University, Shanghai 200433, People's Republic of China; ^2^ Department of Mechanical and Aerospace Engineering, University of California, Los Angeles, CA 90095, USA; ^3^ Key Laboratory of C & PC Structures of Ministry of Education, National Prestress Engineering Research Center, Southeast University, Nanjing 210096, People's Republic of China

**Keywords:** shell theory, buckling, viscoelasticity, pseudo-bistability, snap-through, ellipsoidal shells

## Abstract

Viscoelastic shells subjected to a pressure loading exhibit rich and complex time-dependent responses. Here we focus on the phenomenon of pseudo-bistability, i.e. a viscoelastic shell can stay inverted when pressure is removed, and snap to its natural shape after a delay time. We model and explain the mechanism of pseudo-bistability with a viscoelastic shell model. It combines the small strain, moderate rotation shell theory with the standard linear solid as the viscoelastic constitutive law, and is applicable to shells with arbitrary axisymmetric shapes. As a case study, we investigate the pseudo-bistable behaviour of viscoelastic ellipsoidal shells. Using the proposed model, we successfully predict buckling of a viscoelastic ellipsoidal shell into its inverted configuration when subjected to an instantaneous pressure, creeping when the pressure is held, staying inverted after the pressure is removed, and eventually snapping back after a delay time. The stability transition of the shell from a monostable, temporarily bistable and eventually back to the monostable state is captured by examining the evolution of the instantaneous pressure–volume change relation at different time of the holding and releasing process. A systematic parametric study is conducted to investigate the effect of geometry, viscoelastic properties and loading history on the pseudo-bistable behaviour.

This article is part of the theme issue 'Probing and dynamics of shock sensitive shells'.

## 1. Introduction

Shell buckling has been intensively studied for almost a century [1], and will continuously attract the great attention of researchers in the field of solid mechanics. Not only are shell structures broadly used in various industrial sectors [2–4], but also the analysis of shell buckling can improve the understanding of the motion and morphogenesis of organisms [5,6], which further inspires the design of materials and devices with novel functionalities endowed by the nonlinearity and instability of shells [7,8].

Elastic shells can undergo snap-through buckling when loaded, in which they rapidly reconfigure from one state to another when no stable equilibrium state exists nearby. Such instabilities are ubiquitous in nature and our daily life: Venus flytrap, the famous plant capable of fast movement, can rapidly flip its bistable leaves to catch prey [6], while hair clips can be quickly sprung up and down by fingers. The snap-through buckling of elastic shells has been widely employed to achieve rapid deformation in various applications, such as soft actuators [9–12], logic switches [13,14], and responsive surfaces [15]. Many of these works are based on elastomeric shells, most of which exhibit viscoelasticity. The snap-through buckling of shells could be profoundly influenced by viscoelasticity manifested by creep and stress relaxation. One example is inducing the so-called ‘pseudo-bistable’ behaviour [16–21], which can be illustrated by children's jumping poppers [17]. A jumping popper is a rubber spherical cap that can be buckled into an ‘inside-out’ configuration. After being held for a while and released from the load, the inverted popper undergoes slow creeping for certain delay time, as if it is in a stable equilibrium state, before rapidly snapping back to its natural shape. The recovery time is governed by the viscous time scale of the material and is much longer than that for elastic snap-through buckling. This pseudo-bistable behaviour has been harnessed to achieve spatio-temporal control of morphing structures [16,22–24].

The mechanism of the pseudo-bistability exhibited in buckled viscoelastic shells, however, is far from being clear. The straightforward explanation is that the originally monostable shell can temporarily acquire stability while it is held in its buckled state. When the buckled shell is released, it gradually loses its stability during creeping and eventually snaps back. However, quantitative modelling of viscoelastic shells is essential to support this explanation. One effort is describing a viscoelastic shell by a discrete spring–mass–dashpot system [17,21], where the stability transition is attributed to the change in the ratio of bending to stretching energy caused by viscoelasticity. This argument is not entirely convincing, since it is unclear how the ratio of bending to stretching energy evolves in viscoelastic shells, and how it is related to the stability. Using finite-element simulations together with experiments, researchers capture the pseudo-bistable behaviour of viscoelastic shells [16,21,22]. Although the numerical simulations can accurately predict the responses of viscoelastic shells, they provide limited information on their stability. Recently, Urbach and Efrati proposed a new approach in predicting the stability of viscoelastic solids [20]. In this framework, the behaviour of viscoelastic solids is modelled as an elastic response with respect to a temporally evolving instantaneous reference metric, which determines the stability of the solids. While this approach provides insights into the process of temporarily acquiring and eventually losing stability in the pseudo-bistable behaviour, the understanding of this delayed phenomenon is still unsatisfactory since the instantaneous reference metric is complex and abstract, and moreover, the corresponding surface may not exist in three-dimensional Euclidean spaces.

**Figure 1.**
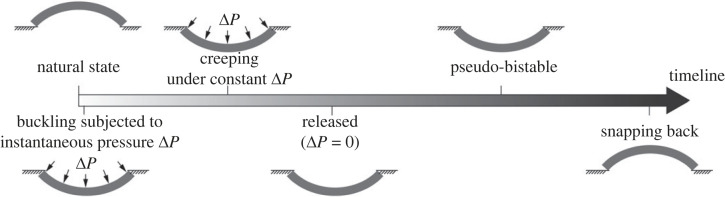
Schematic of the pseudo-bistable behaviour exhibited in viscoelastic shells. A viscoelastic shell buckles into the inverted configuration when an instantaneous pressure Δ*P* is applied. It creeps under the constant Δ*P* for a while before being released. The shell does not recover immediately, but instead, stays inverted as if it were bistable. After a delay time, the shell snaps back to its unbuckled configuration.

In this paper, we aim to model and explain the pseudo-bistability phenomenon by developing a viscoelastic shell model. In our previous work, we have established a shell model for viscoelastic spherical shells [25], based on the small strain, moderate rotation shell theory [26,27] combined with the viscoelastic material law of standard linear solids. Here we further extend the viscoelastic shell model to shells with arbitrary axisymmetric shapes. As a case study, we will investigate the pseudo-bistable behaviour of viscoelastic ellipsoidal shells. Since pseudo-bistability tends to occur in deep shells, which have relatively high strain and rotation when fully inverted, in order to capture the pseudo-bistable behaviour, but at the same time, to limit the deformation within the assumption of small strain and moderate rotation, we will carefully investigate and properly select the geometry, viscoelastic properties and boundary conditions of the shells. Using the proposed shell model, we will predict buckling of a viscoelastic ellipsoidal shell into its inverted configuration when subjected to an instantaneous pressure load, and snapping-through after a delay time when the pressure load is held constantly for a while prior to being removed (figure 1). Moreover, we will use the model to probe the stability of the shell at different times during the holding and releasing process by plotting the corresponding instantaneous pressure–volume change relations. The evolution of the instantaneous pressure–volume change relation confirms the stability transition of the shell from a monostable state, temporarily bistable state and eventually back to the monostable state over time. Finally, the critical creeping time, the minimum time period within which the pressure is held to achieve pseudo-bistability, as well as the recovery time, the delay time before a shell snaps back when the pressure is removed, are predicted using the proposed shell model.

This paper is structured as follows. In §2, a model for viscoelastic shells of arbitrary axisymmetric shapes is formulated by combining the small strain, moderate rotation shell theory with the linearly viscoelastic constitutive relation. The equilibrium equations are derived using the principle of virtual work. In §3, the buckling of ellipsoidal shells of different geometry and viscoelastic properties at different loading rates are investigated to provide insights into the pseudo-bistability of viscoelastic shells. In §4, the pseudo-bistable behaviour is captured by the proposed shell model. The instantaneous pressure–volume change relations at different times during the holding and releasing process are obtained to probe the stability transition. In §5, a parametric study on the critical creeping time and the recovery time is conducted. The conclusion is made in §6.

## 2. Modelling viscoelastic shells with arbitrary axisymmetric shapes

### (a) Small strain, moderate rotation shell theory

**Figure 2.**
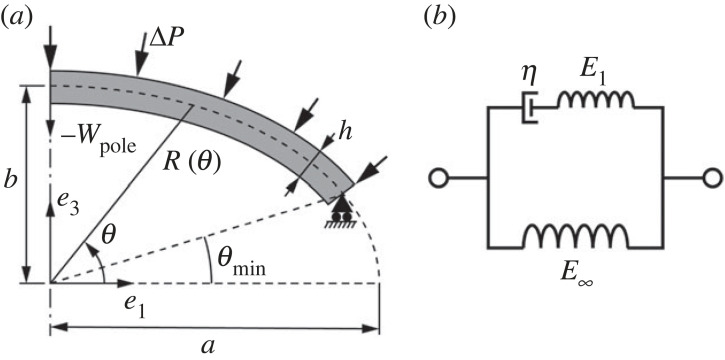
(*a*) Schematic of a viscoelastic shell. The shell with thickness *h* is subjected to a live pressure load Δ*P*. The coordinate *θ* is the meridional angle, ranging from *θ*_min_ to *π*/2. *R*(*θ*) represents the middle surface radius of the undeformed shell. The shell can slide freely along the *e*_1_ axis but has zero displacement along *e*_3_ at *θ* = *θ*_min_, and is subjected to the axisymmetric boundary condition about the *e*_3_ axis at *θ* = *π*/2. (*b*) A standard linear solid containing a Maxwell model with a spring of modulus *E*_1_ and a dashpot of viscosity *η*, connected in parallel to a spring of modulus *E*_∞_.

The schematic of a shell structure with thickness *h* is shown in figure 2*a*. Here we limit ourselves to axisymmetric shells about the *e*_3_ axis. Two surface coordinates (*θ*, *ω*) are used to describe the mid-surface of the shell, in which *θ* is the meridional angle ranging from *θ*_min_ to *π*/2 at the pole, and *ω* is the circumferential angle (not shown in figure 2*a*). The mid-surface radius *R*(*θ*), which quantifies the shape of the shell, could be any smooth function of *θ*.

The small strain, moderate rotation shell theory [26–28] is used to describe the deformation of viscoelastic shells. The position vector ***x*** of a material point with a coordinate (*θ*, *ω*) on the mid-surface of the undeformed shell can be expressed in the three-dimensional Euclidean space as

2.1 $$x(\theta ,\omega )=[R(\theta )\cos\theta \cos\omega ]e_{1}+[R(\theta )\cos\theta \sin\omega ]e_{2}+[R(\theta )\sin\theta ]e_{3},$$

where {*e*_1_, *e*_2_, *e*_3_} is a group of orthonormal bases in the Euclidean space. The displacement of this material point can be written as

2.2 $$\delta (\theta ,\omega )=u^{\beta }x_{,\beta }+wN\text{, }$$

where ***x***_,*β*_ = ∂***x***/∂*β* and ***N*** denote the covariant bases and the normal vector of the mid-surface at the undeformed state, respectively, and (*u^β^*, *w*) are the corresponding displacements. A Greek index takes on values of *θ* and *ω*, and a repeated Greek index means summation over *θ* and *ω*. In this paper, we only consider axisymmetric deformations. As a result, *u^θ^* and *w* are only functions of *θ*, *u^ω^* = 0, and the rotation about ***x***_,*θ*_, *φ_ω_* = *φ^ω^* = 0. The corresponding non-zero mid-surface strains and curvature strains under axisymmetric deformation are [26,27]

2.3 $$\begin{array}{ll} & E_{\omega }^{\omega }=u^{\theta }\Gamma_{\theta \omega }^{\omega }+b_{\omega }^{\omega }w,\\ & E_{\theta }^{\theta }={u^{\theta }}^{\prime }+u^{\theta }\Gamma_{\theta \theta }^{\theta }+b_{\theta }^{\theta }w+\frac{1}{2}\varphi^{2}g_{\theta \theta },\\ & K_{\omega }^{\omega }=\varphi \Gamma_{\theta \omega }^{\omega }\\ \;\text{and}\; & K_{\theta }^{\theta }=\varphi^{\prime }+\varphi \Gamma_{\theta \theta }^{\theta }, \end{array}\}$$

where ${\left(\cdot \right)}^{\prime }$ denotes d(⋅)/dθ, *φ* =* φ^θ^* = *g^θθ^φ_θ_*, where *φ_θ_* denotes the rotation about ***x***_,*ω*_,

2.4 $$\varphi =-w^{\prime }g^{\theta \theta }+b_{\theta }^{\theta }u^{\theta },$$

$\Gamma_{\theta \omega }^{\omega }$ and $\Gamma_{\theta \theta }^{\theta }$ are Christoffel symbols (equation (A 22)), *g_αβ_* and *g^αβ^* are the covariant and contravariant components of the first fundamental form of the mid-surface (equation (A 19)), and $b_{\alpha }^{\beta }$ are the mixed components of the second fundamental form of the mid-surface (equation A 20). Compared to spherical shells, non-spherical shells have much more complex strains in equation (2.3) due to the non-constant *R*(*θ*), since all the coefficients of *u^θ^*, *w*, *φ* and their derivatives depend on *θ*. The mid-surface and curvature strains in equation (2.3) can be expressed in terms of *u^θ^* and *w*, or equivalently in terms of *φ* and *w*. Here we choose *φ* and *w* as the two independent variables by replacing *u^θ^* with a function of *φ* and *w* obtained from equation (2.4). The strain of the shell at an arbitrary position can be expressed as $\varepsilon_{\alpha }^{\beta }=E_{\alpha }^{\beta }+zK_{\alpha }^{\beta }$, where *z* is the coordinate in the thickness direction of the shell and measured from the mid-surface.

### (b) Viscoelastic constitutive relations

Following our previous work [25], here we develop a viscoelastic constitutive relation for viscoelastic shells. We use the Boltzmann superposition principle to quantify the effect of strain history on the current stress state. The two-dimensional stress–strain relation of viscoelasticity under plane stress can be written as

2.5 $$\sigma_{\alpha \beta }(t)=\int_{0}^{t}\frac{E(t-\tau )}{1-\upsilon^{2}}\left[(1-\upsilon )\frac{\text{d}\varepsilon_{\alpha \beta }(\tau )}{\text{d}\tau }+\upsilon \frac{\text{d}\varepsilon_{\gamma }^{\gamma }(\tau )}{\text{d}\tau }g_{\alpha \beta }\right]\text{d}\tau ,$$

where *E*(*t*) is the relaxation modulus as a function of time *t*, υ is the Poisson's ratio, assumed to be a constant, and *α* and *β* are two free indices taking on values *θ* and *ω*. We quantify the material viscoelasticity using the standard linear solid model (figure 2*b*), which contains a Maxwell model with a spring of modulus *E*_1_ and a dashpot of viscosity *η*, connected in parallel to a spring of modulus *E*_∞_. Accordingly, the relaxation modulus takes the following form

2.6 $$E(t)=E_{\infty }+E_{1}{\text{e}}^{-t/\tau_{r}},$$

where *τ_r_* = *η*/*E*_1_ represents the relaxation time.

Integrating the stresses in equation (2.5) and stresses multiplied by distance over the thickness yield the resultant membrane stresses *N_αβ_*(*t*) and the bending moments *M_αβ_*(*t*) at time *t*, respectively,

2.7 $$N_{\alpha \beta }(t)=\int_{-h/2}^{h/2}\sigma_{\alpha \beta }(t)\;\text{d}z,\;\text{and}\;M_{\alpha \beta }(t)=\int_{-h/2}^{h/2}\sigma_{\alpha \beta }(t)z\;\text{d}z.$$

Substituting the non-zero strains in equation (2.3) and the constitutive relation in equation (2.5) into equation (2.7), we obtain the non-zero resultant membrane stresses and the bending moments,

2.8 $$\begin{array}{l} N^{\omega \omega }(t)=\frac{hg^{\omega \omega }}{1-\upsilon^{2}}\int_{0}^{t}E(t-\tau )\left[\frac{\text{d}E_{\omega }^{\omega }(\tau )}{\text{d}\tau }+\upsilon \frac{\text{d}E_{\theta }^{\theta }(\tau )}{\text{d}\tau }\right]\text{d}\tau ,\\ N^{\theta \theta }(t)=\frac{hg^{\theta \theta }}{1-\upsilon^{2}}\int_{0}^{t}E(t-\tau )\left[\frac{\text{d}E_{\theta }^{\theta }(\tau )}{\text{d}\tau }+\upsilon \frac{\text{d}E_{\omega }^{\omega }(\tau )}{\text{d}\tau }\right]\text{d}\tau ,\\ M^{\omega \omega }(t)=\frac{h^{3}g^{\omega \omega }}{12(1-\upsilon^{2})}\int_{0}^{t}E(t-\tau )\left[\frac{\text{d}K_{\omega }^{\omega }(\tau )}{\text{d}\tau }+\upsilon \frac{\text{d}K_{\theta }^{\theta }(\tau )}{\text{d}\tau }\right]\text{d}\tau \\ \;\text{and}\;M^{\theta \theta }(t)=\frac{h^{3}g^{\theta \theta }}{12(1-\upsilon^{2})}\int_{0}^{t}E(t-\tau )\left[\frac{\text{d}K_{\theta }^{\theta }(\tau )}{\text{d}\tau }+\upsilon \frac{\text{d}K_{\omega }^{\omega }(\tau )}{\text{d}\tau }\right]\text{d}\tau . \end{array}\}$$

### (c) Principle of virtual work and equilibrium equations

Following the literature [26,27], here we use the principle of virtual work to derive the equilibrium equations at a given moment *t*. Let *δu_θ_* and *δw* be the virtual displacements of the mid-surface of the shell at time *t*. The associated virtual strains can be expressed as *δ*ϵ*_αβ_* = *δE_αβ_* + *zδK_αβ_*. The internal virtual work (IVW) of the shell is

2.9 $$\text{IVW}=\int_{S}^{}\text{d}S\int_{-h/2}^{h/2}\text{d}z\sigma^{\alpha \beta }\delta \varepsilon_{\alpha \beta }=\int_{S}\left[N^{\alpha \beta }\delta E_{\alpha \beta }+M^{\alpha \beta }\delta K_{\alpha \beta }\right]\;\text{d}S,$$

where *S* represents the area of the mid-surface of the shell. The external virtual work (EVW) due to a uniform live pressure Δ*P* acting on the shell is [26,27] (equation (A 6))

2.10 $$\text{EVW}=\int_{S}[\Delta P\varphi \delta u_{\theta }+\Delta P(1+u_{,\gamma }^{\gamma }+b_{\gamma }^{\gamma }w)\delta w]\;\text{d}S+\oint_{C}\left(T^{\theta }\delta u_{\theta }+Q\delta w-M_{n}\delta w_{,n}\right)\;\text{d}s$$

where *T^θ^* represents the edge resultant traction along ***x***_,*θ*_, *Q* represents the normal edge force, and *M_n_* = *M^αβ^n_α_n_β_* is the component of the edge moment, *n_β_* denotes the components of the unit vector normal to the boundary *C* tangent to the shell, and *s* is the length of the edge of the shell. Enforcing IVW = EVW yields the following equilibrium equations (see Appendix A for details)

2.11 $$\begin{array}{rl} & \;-M_{,\alpha \beta }^{\alpha \beta }+N^{\alpha \beta }b_{\alpha \beta }+{\left(N^{\alpha \beta }\varphi_{\alpha }\right)}_{,\beta }=\Delta P(1+u_{,\gamma }^{\gamma }+b_{\gamma }^{\gamma }w),\\ & \;-N_{,\beta }^{\theta \beta }-M_{,\beta }^{\alpha \beta }b_{\alpha }^{\theta }-\frac{1}{2}{\left(M^{\alpha \beta }b_{\alpha }^{\theta }-M^{\theta \alpha }b_{\alpha }^{\beta }\right)}_{,\beta }+N^{\alpha \beta }\varphi_{\alpha }b_{\beta }^{\theta }=\Delta P\varphi , \end{array}$$

where ()_,_*_α_* and ()_,_*_αβ_* are the first- and second-order covariant derivatives of ().

In equation (2.11), *φ* and *w* are the two independent variables, and the highest order terms are *φ*′′′ and *w*′′′, yielding a system of six-order nonlinear ordinary differential equations (ODEs). In order to limit the deformation within the assumption of small strain and moderate rotation for a deep shell that possesses pseudo-bistability when fully inverted, we choose the sliding boundary for the shell, i.e. on the boundary at *θ* = *θ*_min_, the shell is allowed to slide freely along *e*_1_, but not along *e*_3_ (figure 2*a*). As a result, the traction along *e*_1_ is zero

2.12 $$(T^{\theta }x_{,\theta }+QN)\cdot e_{1}=T^{\theta }(R^{\prime }\cos\theta -R\sin\theta )+Q\frac{R^{\prime }\sin\theta +R\cos\theta }{\sqrt{{R^{\prime }}^{2}+R^{2}}}=0,$$

where *T^θ^* and *Q* are given by equation (A 8), and the displacement along *e*_3_ is zero,

2.13 $$\delta \cdot e_{3}=u^{\theta }(R^{\prime }\sin\theta +R\cos\theta )-\frac{w(R^{\prime }\cos\theta -R\sin\theta )}{\sqrt{{R^{\prime }}^{2}+R^{2}}}=0.$$

In addition, the assumption of axisymmetric deformation requires *w*′= *φ* = *φ*′′= 0 at the pole (θ=π/2).

φ and *w* at time *t* can be obtained by solving the above boundary value problem, using the bvp4c solver and the finite difference method in Matlab. Here we consider three types of loading: (i) pressure-controlled loading, (ii) displacement-controlled loading and (iii) volume-controlled loading. When the pressure Δ*P* serves as the load parameter, the equilibrium equations in equation (2.11) are solved with prescribed evolution of pressure as a function of time. Displacement-controlled loading means that the displacement at the pole $w_{\text{pole}}=w(\theta =\pi /2)$ (figure 2*a*) is prescribed as the load parameter. With this loading type, the pressure is treated as an extra variable. Correspondingly, an additional ODE, Δ*P*^′^ = 0, is added to the ODE set. Volume-controlled loading is achieved by setting the volume of the shell as an additional variable and adding an extra constraining ODE relating the volume and displacement to the ODE set, where the pressure is regarded as an extra variable as well. In this paper, the pressure-controlled loading is used to demonstrate the pseudo-bistable behaviour while the volume-controlled loading is used to produce the instantaneous pressure-volume change relations to examine the stability transition during the pseudo-bistability phenomenon. Due to the low practicality in operation, the displacement-controlled loading is only used to assist finding the equilibrium pressure-volume change paths for some elastic shells, especially for those exhibiting unstable paths when the other two loading types are adopted.

## 3. Rate-dependent buckling behaviours

In §2, we establish a model for viscoelastic shells with arbitrary axisymmetric shapes. In this section, we will study particular examples of ellipsoidal shells with the following *R*(*θ*)

3.1 $$R(\theta )=\frac{b}{\sqrt{1-e^{2}\;\cos^{2}\theta }},$$

where $e=\sqrt{1-{\left(b/a\right)}^{2}}$ denotes eccentricity, and *a* and *b* denote the half lengths of the major and minor axes, respectively. Using the proposed shell model, we will first conduct the buckling analysis of elastic shells to figure out how geometry influences the stability of the shells. We then examine the effect of loading rates and viscoelastic properties on the rate-dependent buckling behaviour of viscoelastic shells. The analysis in this section provides insights into choosing proper geometric parameters and material properties to achieve pseudo-bistability in viscoelastic shells.

### (a) Buckling of elastic shells

**Figure 3.**
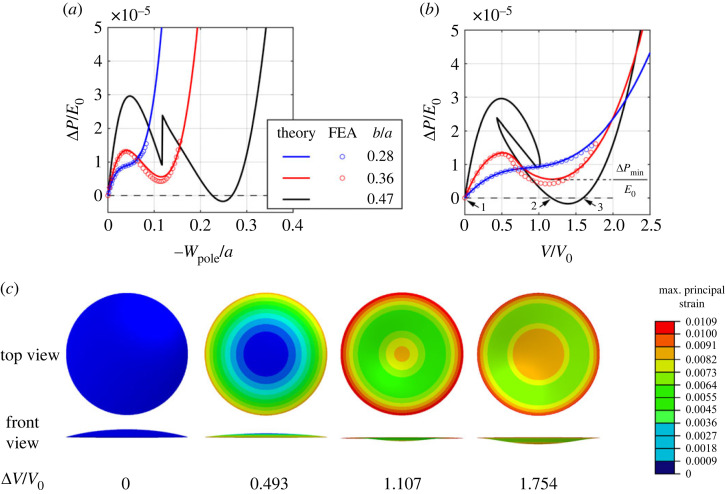
Buckling behaviour of elastic ellipsoidal shells with *h*/*a* = 0.02, *θ*_min_ = 17π/128, and different *b*/*a*. (*a*) Normalized pressure Δ*P*/*E*_0_ versus normalized displacement − *w*_pole_/*a* at the pole. (*b*) Normalized pressure Δ*P*/*E*_0_ versus normalized volume change Δ*V*/*V*_0_, where *V*_0_ denotes the negative volume of the undeformed shell. The solid curves represent the theoretical results for the shells with *b*/*a* = 0.28, 0.36 and 0.47, and the circular dots represent the FEA results for the shell with *b*/*a* = 0.28 and 0.36. The Δ*P*/*E*_0_–Δ*V*/*V*_0_ curve for *b*/*a* = 0.47 intersects with the horizontal line of Δ*P* = 0 (dashed line) at three points, indicating that the shell with *b*/*a* = 0.47 is bistable. (*c*) The deformed shapes obtained from FEA for the shell with *b*/*a* = 0.36 at different volume changes are axisymmetric. The contour represents the maximum principal strain. (Online version in colour.)

The buckling behaviour of elastic ellipsoidal shells subjected to uniform live pressures can be obtained by solving the equilibrium equations (equation (2.11)) and boundary conditions (equations (2.12), (2.13)) with the following isotropic linearly elastic material law,

3.2 $$\begin{array}{l} \;N^{\alpha \beta }=\frac{E_{0}h}{1-\nu^{2}}[(1-\upsilon )E^{\alpha \beta }+\upsilon E_{\gamma }^{\gamma }g^{\alpha \beta }]\\ \;\text{and}\;M^{\alpha \beta }=\frac{E_{0}h^{3}}{12(1-\nu^{2})}[(1-\upsilon )K^{\alpha \beta }+\upsilon K_{\gamma }^{\gamma }g^{\alpha \beta }], \end{array}\}$$

where *E*_0_ denotes the Young's modulus, and υ is the Poisson's ratio, which is assumed to equal 0.5 (incompressible material) throughout this paper. Figure 3 shows the relations between the normalized pressure Δ*P*/*E*_0_ and normalized displacement − *w*_pole_/*a* at the pole (figure 3*a*), as well as the relations between the normalized pressure Δ*P*/*E*_0_ and normalized volume change Δ*V*/*V*_0_ (figure 3*b*) for elastic ellipsoidal shells with *h*/*a* = 0.02, *θ*_min_ = 17*π*/128, and different minor-to-major-length ratios *b*/*a* quantifying the shallowness of the shells. Here Δ*V* is the volume change of the shell with respect to the undeformed state at *t* = 0, and *V*_0_ represent the negative volume of the shell in the undeformed state,

3.3 $$V_{0}=-\int_{\theta_{\text{min}}}^{\pi /2}\pi R^{2}\cos^{2}\theta (R^{\prime }\sin\theta +R\cos\theta )\;\text{d}\theta .$$

The shells with different minor-to-major-length ratios *b*/*a* exhibit quite different Δ*P*/*E*_0_–Δ*V*/*V*_0_ curves (figure 3*b*). When *b*/*a* = 0.28, Δ*P*/*E*_0_ increases monotonically with the increase of Δ*V*/*V*_0_. For *b*/*a* = 0.36, Δ*P*/*E*_0_ initially increases, then decreases after the shell buckles, and increases again with the increase of Δ*V*/*V*_0_, showing the features of snap-through buckling. The above two shells are monostable, since their Δ*P*/*E*_0_ remains positive. As *b*/*a* becomes large (*b*/*a* = 0.47), both Δ*P*/*E*_0_ and Δ*V*/*V*_0_ change non-monotonically, forming a very complex curve. Moreover, the Δ*P*/*E*_0_–Δ*V*/*V*_0_ curve intersects with the horizontal line of Δ*P* = 0 (dashed line) at three points (figure 3*b*), where points 1 and 3 represent two stable equilibrium states while point 2 represents an unstable equilibrium state when Δ*P* = 0. Therefore, the three intersection points indicate that the shell with *b*/*a* = 0.47 is bistable. The stability of the shells can also be measured by the local minimum pressure Δ*P*_min_/*E*_0_ of the Δ*P*/*E*_0_–Δ*V*/*V*_0_ curve. A positive Δ*P*_min_/*E*_0_ means monostability, whereas a negative Δ*P*_min_/*E*_0_ indicates that the shell has more than one stable state when Δ*P* = 0, and therefore bistability. From figure 3 we can see that the stability of shells (monostability or bistability) can be tuned by the minor-to-major-length ratio *b*/*a*: a deeper shell with a higher *b*/*a* is more likely to be bistable. The Δ*P*/*E*_0_–Δ*V*/*V*_0_ curves for *b*/*a* = 0.28 and 0.36 can be obtained by prescribing a monotonic increase in the load parameter of either *w*_pole_/*a* or Δ*V*/*V*_0_. The two loading methods yield the exact same Δ*P*/*E*_0_–Δ*V*/*V*_0_ curves. However, the Δ*P*/*E*_0_–Δ*V*/*V*_0_ equilibrium path for *b*/*a* = 0.47 can only be achieved by treating *w*_pole_/*a* as the load parameter, since *w*_pole_/*a* rather than Δ*V*/*V*_0_ or $\Delta P/\mu_{0}$ increases monotonically along the equilibrium path.

To verify the axisymmetric deformation of the shells with the chosen geometry, we conduct finite-element analysis (FEA) for the shells without axisymmetric constraints using the commercial software Abaqus/Standard. The static Riks method is implemented to capture the unstable equilibrium Δ*P*/*E*_0_–Δ*V*/*V*_0_ curve of the elastic ellipsoidal shells under pressure-controlled loading and the boundary condition as shown in equations (2.12)(2.13). The shells are modelled as an incompressible linearly elastic material with 8-node doubly curved thick shell elements with reduced integration (Abaqus type S8R). We plot the Δ*P*/*E*_0_–Δ*V*/*V*_0_ curves from FEA (circular dots in figure 3*a,b*) for the shells with *b*/*a* = 0.28 and 0.36. We find very good agreement between the results from the shell model and FEA for *b*/*a* = 0.28, and the deformation of the shell in FEA is also axisymmetric. For *b*/*a* = 0.36, although there is slight deviation between the results from the shell model and FEA after the buckling, the Δ*P*/*E*_0_–Δ*V*/*V*_0_ curves obtained from the shell model can still reasonably capture the deformation process. Therefore, the shell model is still a good analytical tool for us to understand the mechanism of pseudo-bistaiblity. Moreover, the deformation of the shell with *b*/*a* = 0.36 in FEA is also axisymmetric. In figure 3*c* we plot the deformed shapes of the shell with *b*/*a* = 0.36 when it is on the edge of buckling ($\Delta V/V_{0}=0.493$), the pressure reaches a local minimum ($\Delta V/V_{0}=1.107$), and the shell is fully inverted ($\Delta V/V_{0}=1.754$). The deformation mode for *b*/*a* = 0.47 from FEA, however, is no longer axisymmetric. Therefore, in the following, we will limit ourselves to shells with *b*/*a* ≤ 0.36.

### (b) Buckling of viscoelastic shells

Next, we will examine the buckling behaviours of viscoelastic ellipsoidal shells under volume-controlled loading over a wide range of loading rates. The influence of the relative modulus of relaxation, *E*_rel_ = *E*_1_/*E*_0_, which is the ratio of the modulus in the Maxwell element *E*_1_ to the instantaneous modulus *E*_0_ = *E*_1_ + *E*_∞_, on the buckling behaviour is also studied. We define a dimensionless loading rate, *γ_V_*, to quantify the rate of changes in the volume,

3.4 $$\gamma_{V}=\frac{\text{d}(\Delta V/V_{0})}{\text{d}(t/\tau_{r})},$$

which indicates that in the relaxation time scale *τ_r_* the volume change is *V*_0_*γ_V_*. In the following we will take the shell with *b*/*a* = 0.36 as an example and study its rate-dependent buckling behaviour. Other geometric parameters are *h*/*a* = 0.02, *θ*_min_ = 17*π*/128.

**Figure 4.**
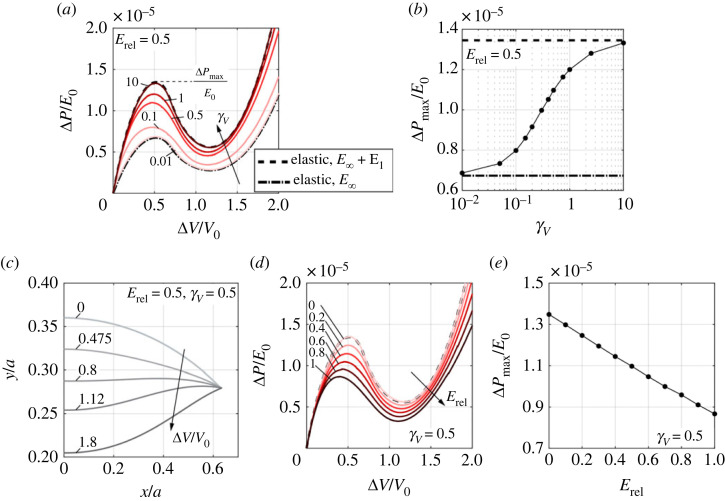
Buckling behaviours of viscoelastic shells with *b*/*a* = 0.36 under volume-controlled loading. (*a*) Normalized pressure Δ*P*/*E*_0_–volume change Δ*V*/*V*_0_ relations at different volume loading rates $\left(0.01\leq \gamma_{V}\leq 10\right)$ when *E*_rel_ = 0.5. (*b*) Buckling pressure Δ*P*_max_/*E*_0_ as a function of *γ_V_*. The dashed and dot-dashed lines represent Δ*P*_max_/*E*_0_ for elastic shells with moduli *E*_∞_ + *E*_1_ and *E*_∞_, respectively. (*c*) Middle-surface profiles of the shell under different volume changes Δ*V*/*V*_0_ when *E*_rel_ = 0.5 and *γ_V_* = 0.5. (*d*) Normalized pressure Δ*P*/*E*_0_–volume change Δ*V*/*V*_0_ relations at different relative modulus of relaxation $\left(0\leq E_{\text{rel}}\leq 1\right)$ when *γ_V_* = 0.5. (*e*) Buckling pressure Δ*P*_max_/*E*_0_ as a function of *E*_rel_. The dashed and dot-dashed lines in (*a*) and (*d*) represent the pressure–volume change relations for elastic shells with moduli *E*_∞_ + *E*_1_ and *E*_∞_, respectively. (Online version in colour.)

We first examine the influence of the loading rates on the Δ*P*/*E*_0_–Δ*V*/*V*_0_ curve under volume-controlled loading. The curves corresponding to different volume loading rates *γ_V_* ranging from 0.01 to 10 are plotted in figure 4*a* when the relative modulus of relaxation *E*_rel_ is fixed at 0.5. When *γ_V_* is very low (*γ_V_* = 0.01), almost full relaxation occurs, and the response of the shell is governed by the long-term modulus, *E*_∞_. As a result, the Δ*P*/*E*_0_–Δ*V*/*V*_0_ curves at very low *γ_V_* approach that of the elastic shell with modulus *E* = *E*_∞_ (dot-dashed line in figure 4*a*). On the other hand, the very high *γ_V_* (*γ_V_* = 10) results in little relaxation. Correspondingly, the effective modulus of the shell is close to the instantaneous modulus *E*_0_ = *E*_1_ + *E*_∞_, and thus the Δ*P*/*E*_0_–Δ*V*/*V*_0_ curves at very high *γ_V_* approach that of the elastic shell with modulus *E*_0_ = *E*_1_ + *E*_∞_ (dashed line in figure 4*a*). The Δ*P*/*E*_0_–Δ*V*/*V*_0_ curves at moderate *γ_V_* are located in between the two extreme cases, and the resultant pressure Δ*P*/*E*_0_ for a given volume change Δ*V*/*V*_0_ increases as *γ_V_* increases. The buckling pressure $\Delta P_{\text{max}}/E_{0}$ at very high (low) *γ_V_* approaches that of the elastic shell with *E*_1_ + *E*_∞_ (*E*_∞_) (figure 4*b*). In between the very low and very high *γ_V_*, the increase of *γ_V_* results in a notable increase in $\Delta P_{\text{max}}/E_{0}$ (figure 4*b*). The middle-surface profiles of the shell under different volume changes Δ*V*/*V*_0_ at the volume loading rate *γ_V_* = 0.5 is shown in figure 4*c*. The profile of the shells stays concave before the pressure reaches the critical pressure for buckling when $\Delta V/V_{0}=0.475$, and transitions from concave to convex as the pressure decreases and the volume change increases ($0.475<\Delta V/V_{0}<1.12$). Finally, the profile keeps convex while the pressure increases again with the increase of the volume change ($\Delta V/V_{0}\geq 1.12$).

The Δ*P*/*E*_0_–Δ*V*/*V*_0_ curves for viscoelastic shells also highly depend on the relative modulus of relaxation, *E*_rel_. We consider a moderate loading rate *γ_V_* = 0.5, and plot the Δ*P*/*E*_0_–Δ*V*/*V*_0_ curves for different *E*_rel_ ranging from 0 to 1, as shown in figure 4*d*. When *E*_rel_ = 0, no relaxation occurs, and thus the corresponding Δ*P*/*E*_0_–Δ*V*/*V*_0_ curve coincides with that of the elastic shell with modulus *E*_0_ = *E*_1_ + *E*_∞_ (dashed line in figure 4*d*). As *E*_rel_ increases from 0, the resultant pressure Δ*P*/*E*_0_ reduces notably for a given volume change Δ*V*/*V*_0_, leading to a reduction in $\Delta P_{\text{max}}/E_{0}$ (figure 4*e*).

## 4. Mechanism of pseudo-bistability

In this section, we will first use the viscoelastic shell model formulated in Section 2 to demonstrate the pseudo-bistability phenomenon, in which an inverted viscoelastic ellipsoidal shell snaps back to its natural state with a delay time after a pressure load is held constantly for a while prior to being released. We will then probe the stability of the shell at different time during this holding and releasing process by plotting the corresponding instantaneous pressure–volume change relations. The obtained stability transition provides insights into the mechanism of the pseudo-bistability.

### (a) Predicting pseudo-bistable behaviour

**Figure 5.**
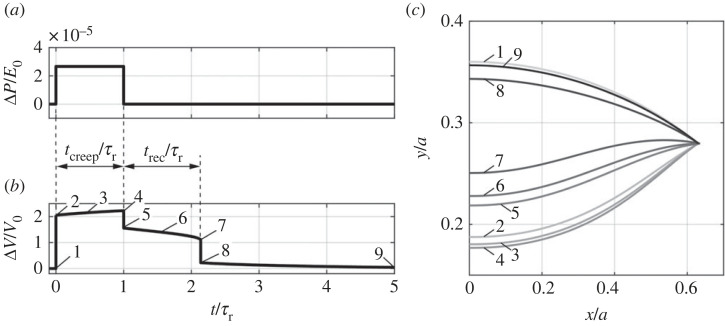
Pseudo-bistable behaviour of a viscoelastic shell. (*a*) Applied pressure-time relation and (*b*) the corresponding volume change-time relation for a viscoelastic ellipsoidal shell with *b*/*a* = 0.36, *h*/*a* = 0.02, *θ*_min_ = 17*π*/128 and *E*_rel_ = 0.5. The time period within which a constant pressure is held is defined as the creeping time *t*_creep_, and the time period within which the shell stays inverted after the pressure is removed is defined as the recovery time *t*_rec_. *τ*_r_ = *η*/*E*_1_ denotes the relaxation time constant of the viscoelastic material. (*c*) Middle-surface profiles of the shell at different time moments as labelled in (*b*).

Demonstrating pseudo-bistability in a viscoelastic shell requires a careful choice of geometry and viscoelastic properties. The geometry should result in a monostable pressure Δ*P*/*E*_0_-volume change Δ*V*/*V*_0_ relation if the shell were elastic, but the minimum normalized pressure $\Delta P_{\text{min}}/E_{0}$ is not too far away from zero. On the other hand, the viscoelastic effects should be large enough to trigger pseudo-bistability. We choose a viscoelastic shell with the geometric parameters as *b*/*a* = 0.36, *h*/*a* = 0.02, *θ*_min_ = 17*π*/128, which corresponds to a monostable shell if it were elastic, and material parameter *E*_rel_ = 0.5. We apply an instantaneous pressure load $\Delta P/E_{0}=2.67\times 10^{-5}$, which is above its buckling pressure $\Delta P_{\text{max}}/E_{0}$, and release this pressure after holding it for *t*_creep_ = *τ_r_* (figure 5*a*). The corresponding volume change Δ*V*/*V*_0_ as a function of time *t* is computed based on the proposed shell model (figure 5*b* and Supplementary video [29]). We observe that the shell immediately buckles into an inverted shape once the pressure is applied (figure 5*b,c*, moment 2), and creeps with a small increase in volume change for *t*_creep_ = *τ_r_* (figure 5*b,c*, from moment 2 to moment 4). After the pressure is removed, the viscoelastic shell can temporarily stay inverted for *t*_rec_ = 1.136*τ_r_* (figure 5*b,c*, from moment 5 to moment 7). At moment 7, a solution of the inverted state can no longer be found using the solution of the last iteration as the initial guess with the ODE solver, but only a solution of the unbuckled state can be found using the undeformed configuration as the initial guess. Accordingly, the shell snaps from the inverted configuration (moment 7) back to the unbuckled configuration (moment 8). After this snapping deformation, the shell gradually recovers its undeformed shape, with Δ*V*/*V*_0_ slowly decreasing to zero. The characteristics of this observed deformation history agree with those of FEA simulations and experiments reported in literature [16,21,22], indicating that the proposed viscoelastic shell model can capture the pseudo-bistability exhibited in viscoelastic shells.

### (b) Stability transition during delayed snap-through

Having successfully predicted the pseudo-bistable behaviour of a viscoelastic shell using the proposed shell model, we next investigate the stability transition of the shell during this holding and releasing process. For an elastic shell, the number of the intersection points of its pressure–volume change curve with the horizontal line of zero pressure determines its stability (figure 3*b*). One intersection point indicates that the shell is monostable, since there is only one stable equilibrium state when no pressure is applied. Three intersection points, on the other hand, indicate that the shell is bistable, since there are two stable and one unstable equilibrium states at zero pressure. To probe the stability evolution of the viscoelastic shell during the hold and releasing process, we need to plot the instantaneous pressure-volume change relation at different time moments and check the number of intersection points with the horizontal line of zero pressure. In §3, we have learned that an extremely fast loading *γ_V_* ≫ 1 can eliminate the viscoelastic relaxation effects and yield an instantaneous pressure-volume change response of a shell. Therefore, in the following we will conduct volume-controlled loading to different time moments of interest in the holding and releasing process, and unload (or load for some cases) at an extremely high rate to obtain the corresponding instantaneous pressure–volume change responses, which provide information on the stability evolution of the viscoelastic shell.

**Figure 6.**
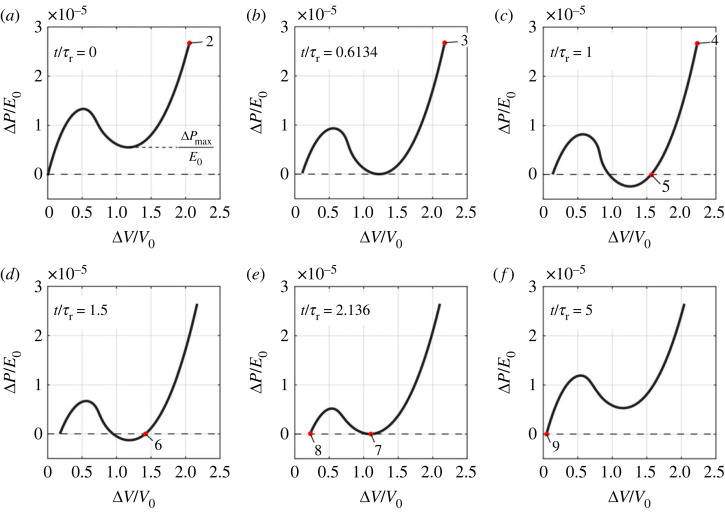
(*a–f*) The instantaneous pressure–volume change relations at the time moments labelled in figure 5*b*. The red dots represent the states of the shells for the corresponding time moments. (Online version in colour.)

We follow the same loading process up to the different time moments as in figure 5*a*, and unload at a very high rate of changes in the volume *γ_V_* = 10 to obtain the instantaneous pressure Δ*P*/*E*_0_-volume change Δ*V*/*V*_0_ relations (figure 6*a–f*). When the shell is unloaded at moment 2, right after the instantaneous pressure is applied (figure 5*a*), the instantaneous Δ*P*/*E*_0_-Δ*V*/*V*_0_ relation (figure 6*a*) is exactly the same as the Δ*P*/*E*_0_-Δ*V*/*V*_0_ curve for the elastic shell with the same geometry (the red curve in figure 3*b*). This agreement is due to the fact that creeping has not yet started and thus viscoelasticity plays no role. At moment 2, the local minimum pressure of the instantaneous Δ*P*/*E*_0_–Δ*V*/*V*_0_ curve, $\Delta P_{\text{min}}/E_{0}$, is larger than zero, so the shell is monostable. When the pressure is held until moment 3, $\Delta P_{\text{min}}/E_{0}$ of the instantaneous Δ*P*/*E*_0_–Δ*V*/*V*_0_ curve decreases to zero (figure 6*b*). When the pressure is held for an even longer time, for example until moment 4, $\Delta P_{\text{min}}/E_{0}$ of the instantaneous Δ*P*/*E*_0_–Δ*V*/*V*_0_ curve becomes negative (figure 6*c*). Accordingly, the number of the intersection points between the instantaneous Δ*V*/*V*_0_–Δ*V*/*V*_0_ curve and the horizontal line of $\Delta P/E_{0}=0$ (dashed line) changes from one (figure 6*a*) to two (figure 6*b*), and eventually to three (figure 6*c*). Thus, the stability of the shell transitions from a monostable state to bistable state due to viscoelastic creeping, with moment 3 as the critical transition time, at which $\Delta P_{\text{min}}/E_{0}=0$.

Right after the pressure is removed, the shell jumps from the configuration at moment 4 to the closest stable configuration (moment 5 in figure 5*b*), which corresponds to the third intersection point between the instantaneous Δ*P*/*E*_0_–Δ*V*/*V*_0_ curve and the horizontal line of $\Delta P/E_{0}=0$ (point 5 in figure 6*c*). This jump results in a sudden drop in Δ*V*/*V*_0_ (from moment 4 to 5 in figure 5*b*). After the load is released ($\Delta P/E_{0}=0$), the instantaneous Δ*P*/*E*_0_–Δ*V*/*V*_0_ curve further evolves: $\Delta P_{\text{min}}/E_{0}$ starts to increase (figure 6*d,e*), and the third intersection point gradually shifts to the left (from point 5 in figure 6*c* to point 6 in figure 6*d*), resulting in a slow decrease in Δ*V*/*V*_0_ (from moment 5 to 6 in figure 5*b*). The shell is bistable and stays inverted as long as $\Delta P_{\text{min}}/E_{0}<0$. When $\Delta P_{\text{min}}/E_{0}$ increases back to zero at moment 7 (figure 6*e*), the third and the second intersection points merge into a single point (point 7 in figure 6*e*) tangent to the horizontal line of $\Delta P/E_{0}=0$. The shell at moment 7 is unstable and thus snaps to the only stable configuration (point 8 in figure 6*e*). Correspondingly, the shell snaps from the inverted state (moment 7) to unbuckled state (moment 8). Therefore, moment 7 is the critical moment at which the stability transitions from the bistable state back to the monostable state. As the creeping process continues, $\Delta P_{\text{min}}/E_{0}$ keeps increasing. As a result, only one intersection point exists and shifts to the left (from point 8 in figure 6*e* to point 9 in figure 6*f*), leading to a decrease in Δ*V*/*V*_0_ (from moment 8 to 9 in figure 5*b*). At moment 9, the instantaneous Δ*P*/*E*_0_–Δ*V*/*V*_0_ curve almost recovers the one at moment 2, the intersection point almost overlaps the origin, and the shell almost recovers the stress-free shape and volume (figure 6*f*).

**Figure 7.**
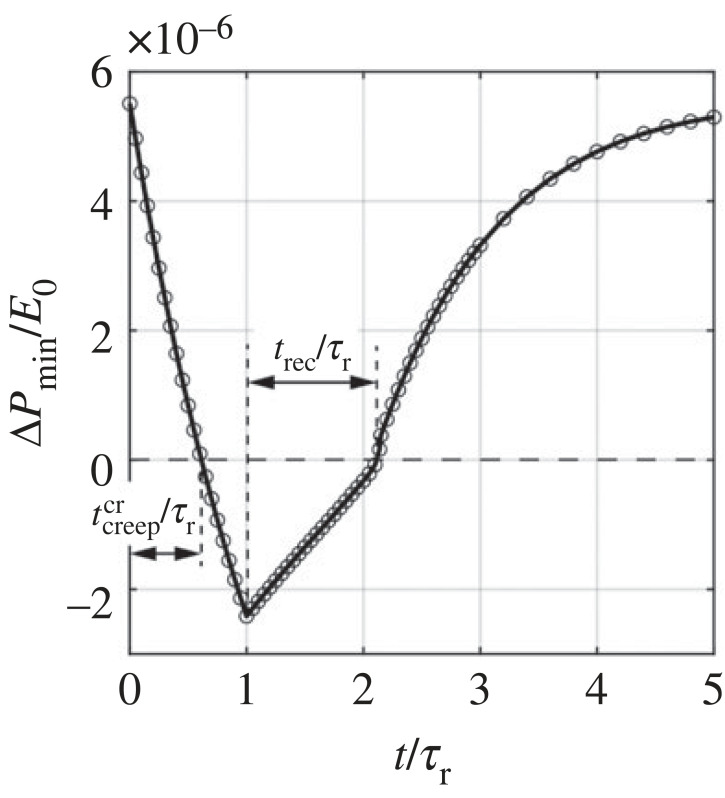
Local minimum pressure Δ*P*_min_/*E*_0_ of the instantaneous pressure–volume change curve as a function of time. The time period within which Δ*P*_min_/*E*_0_ decreases to zero is defined as the critical creeping time $t_{\text{creep}}^{\text{cr}}$, indicating the minimum creeping time required for pseudo-bistability.

We summarize the $\Delta P_{\text{min}}/E_{0}$–time relation in figure 7, from which we can clearly observe the stability transition of the viscoelastic shell from a monostable state to a bistable state, and back to the monostable state during the holding and releasing process. $\Delta P_{\text{min}}/E_{0}$, starting with a positive value, monotonically decreases while the pressure is held constantly, and reaches its minimum when the pressure is removed at *t*/*τ*_r_ = *t*_creep_/*τ*_r_ = 1. Accordingly, the shell is initially monostable, and switches to bistable when $\Delta P_{\text{min}}/E_{0}$ flips its sign from positive to negative, and stays bistable. Here we define the time period within which $\Delta P_{\text{min}}/E_{0}$ decreases to zero as the critical creeping time $t_{\text{creep}}^{\text{cr}}$, representing the minimum creeping time required for the stability transition. Only if the pressure is held for a time period longer than $t_{\text{creep}}^{\text{cr}}$ can the shell exhibit pseudo-bistability. After the shell is released at *t*/*τ*_r_ = 1, $\Delta P_{\text{min}}/E_{0}$ starts to increase. When $\Delta P_{\text{min}}/E_{0}$ flips its sign back to positive, the acquired stability is lost and the shell recovers the monostable state, triggering snapping from the inverted configuration to the unbuckled configuration. The time period within which $\Delta P_{\text{min}}/E_{0}$ increases from its minimum to zero is the recovery time *t*_rec_ defined in figure 5*b*. As time goes on, $\Delta P_{\text{min}}/E_{0}$ continues increasing and approaches its initial value.

## 5. Influence of geometry, viscoelastic property and loading history on pseudo-bistability

In this section, we will investigate how the geometry and viscoelastic property of ellipsoidal shells, and the loading history influence their pseudo-bistable behaviour. Specifically, the minor-to-major-length ratio *b*/*a*, relative modulus of relaxation *E*_rel_, and the holding time *t*_creep_/*τ*_r_ are considered. A higher *b*/*a* (a deeper shell) results in a smaller local minimum pressure $\Delta P_{\text{min}}/E_{0}$ in the instantaneous Δ*P*/*E*_0_-Δ*V*/*V*_0_ curve (figure 3*b*), and thus leads to a shell closer to a bistable one if it were elastic. A larger *E*_rel_ causes a stronger viscoelastic effect, while a longer *t*_creep_/*τ*_r_ results in a longer creeping process. Other parameters such as *h*/*a* = 0.02, *θ*_min_ = 17*π*/128, and the applied pressure $\Delta P/E_{0}=2.67\times 10^{-5}$ are fixed. The critical creeping time $t_{\text{creep}}^{\text{cr}}$ and the recovery time *t*_rec_ will be investigated with respect to different values of the parameters mentioned above.

**Figure 8.**
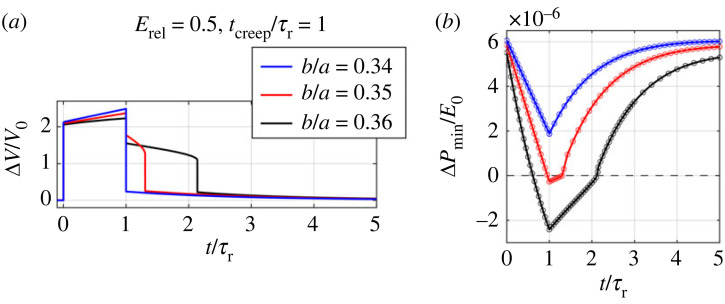
(*a*) Volume change Δ*V*/*V*_0_-time *t*/*τ*_r_ relations and (*b*) local minimum pressure Δ*P*_min_/*E*_0_ of the instantaneous pressure-volume change curves as a function of time *t*/*τ*_r_ for viscoelastic ellipsoidal shells with relative modulus of relaxation *E*_rel_ = 0.5 and different minor-to-major-length ratios *b*/*a* during the holding for *t*_creep_/*τ*_r_ = 1 and releasing process. (Online version in colour.)

We first examine the effect of the minor-to-major-length ratio *b*/*a* on the pseudo-bistable behaviour. We apply an instantaneous pressure and release this pressure after holding it for *t*_creep_/*τ*_r_ = 1 (figure 5*a*). The volume change Δ*V*/*V*_0_ as a function of time *t*, as well as the local minimum pressure $\Delta P_{\text{min}}/E_{0}$ of the instantaneous Δ*P*/*E*_0_-Δ*V*/*V*_0_ curve as a function of time *t* for shells with relative modulus of relaxation *E*_rel_ = 0.5 and different *b*/*a* are plotted in figure 8. From the Δ*V*/*V*_0_-*t* curves (figure 8*a*), we find that the shells with *b*/*a* = 0.35 and 0.36 exhibit pseudo-bistability while the shell with *b*/*a* = 0.34 does not, and that the shell with *b*/*a* = 0.36 has a longer delay time than the one with 0.35. This is because $\Delta P_{\text{min}}/E_{0}$ decreases slower for a shallower shell (lower *b*/*a*) (figure 8*b*) during the holding process. At *t*/*τ*_r_ = *t*_creep_/*τ*_r_ = 1, the shells with *b*/*a* = 0.35 and 0.36 reach negative $\Delta P_{\text{min}}/E_{0}$, indicating that they are temporally bistable. The shell with *b*/*a* = 0.36 has a smaller $\Delta P_{\text{min}}/E_{0}$ than the one with *b*/*a* = 0.35. Thus, it takes longer for the $\Delta P_{\text{min}}/E_{0}$ of the shell with *b*/*a* = 0.36 to recover a positive value after the pressure is removed, leading to a longer recovery time *t*_rec_. In addition, the critical creeping time $t_{\text{creep}}^{\text{cr}}$, the intersection point between the $\Delta P_{\text{min}}/E_{0}$–*t* curve and the horizontal line of $\Delta P_{\text{min}}/E_{0}=0$ (dashed line in figure 8*b*), for *b*/*a* = 0.36 is smaller than the one for *b*/*a* = 0.35. The $\Delta P_{\text{min}}/E_{0}$ for the shell with *b*/*a* = 0.34, however, remains positive at *t*/*τ*_r_ = *t*_creep_/*τ*_r_ = 1, indicating that it stays monostable during the holding process and thus immediately snaps back after it is released. The shell with *b*/*a* = 0.34 needs longer *t*_creep_/*τ*_r_ to reduce $\Delta P_{\text{min}}/E_{0}$ to a negative value in order to trigger pseudo-bistability. For all the three shells, their $\Delta P_{\text{min}}/E_{0}$ recovers the initial values as *t* approaches 5*τ*_r_.

**Figure 9.**
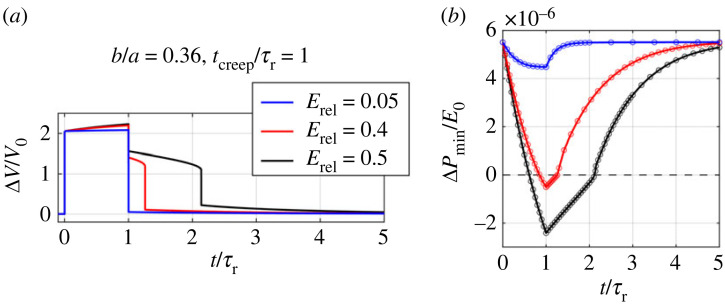
(*a*) Volume change Δ*V*/*V*_0_-time *t*/*τ*_r_ relations and (*b*) local minimum pressure Δ*P*_min_/*E*_0_ of the instantaneous pressure-volume change curves as a function of time *t*/*τ*_r_ for viscoelastic ellipsoidal shells with minor-to-major-length ratio *b*/*a* = 0.36 and different relative moduli of relaxation *E*_rel_ during the holding for *t*_creep_/*τ*_r_ = 1 and releasing process. (Online version in colour.)

We then investigate how the viscoelastic effect influences the pseudo-bistable behaviour. We apply the same loading process as shown in figure 5*a*, and plot the Δ*V*/*V*_0_–*t*/*τ*_r_ curves (figure 9*a*) and corresponding $\Delta P_{\text{min}}/E_{0}$–*t*/*τ*_r_ relations (figure 9*b*) for viscoelastic shells with *b*/*a* = 0.36 and different *E*_rel_. Figure 9*a* shows that the shells with high (*E*_rel_ = 0.5) and intermediate (*E*_rel_ = 0.4) viscoelastic effects exhibit pseudo-bistable behaviour while the shell with a low viscoelastic effect (*E*_rel_ = 0.05) snaps back immediately after the pressure is removed. In addition, a higher viscoelastic effect results in a longer recovery time *t*_rec_. In figure 9*b*, we can clearly see that the stability transitions from a monostable state ($\Delta P_{\text{min}}/E_{0}>0$) to a bistable state ($\Delta P_{\text{min}}/E_{0}<0$) for the shells with *E*_rel_ = 0.4 and 0.5, whereas the shell with *E*_rel_ = 0.05 remains monostable during the holding process. The $\Delta P_{\text{min}}/E_{0}$ for the shell with *E*_rel_ = 0.05 reduces more and more slowly as *t* increases and reaches a plateau at *t*/*τ*_r_ = *t*_creep_/*τ*_r_ = 1, meaning that increasing the holding time can never reduce $\Delta P_{\text{min}}/E_{0}$ to a negative value, and thus leads to no pseudo-bistable behaviour. Therefore, there exists a critical value of *E*_rel_ for shells to achieve pseudo-bistability.

**Figure 10.**
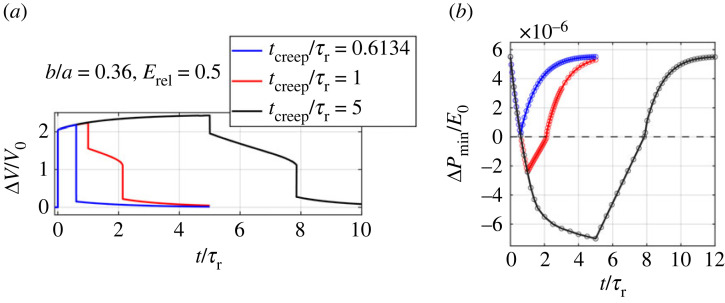
(*a*) Volume change Δ*V*/*V*_0_-time *t*/*τ*_r_ relations and (*b*) local minimum pressure Δ*P*_min_/*E*_0_ of the instantaneous pressure-volume change curves as a function of time *t*/*τ*_r_ for viscoelastic ellipsoidal shells with minor-to-major-length ratio *b*/*a* = 0.36 and relative modulus of relaxation *E*_rel_ = 0.5 during the holding for different *t*_creep_/*τ*_r_ and releasing process. (Online version in colour.)

Moreover, we study the effect of time period of the holding process, *t*_creep_/*τ*_r_, on the pseudo-bistable behaviour of viscoelastic shells. We fix *b*/*a* = 0.36 and *E*_rel_ = 0.5, and vary *t*_creep_/*τ*_r_ (figure 5*a*) from 0.6134, 1 to 5, where *t*_creep_/*τ*_r_ = 0.6134 is the critical creeping time $t_{\text{creep}}^{\text{cr}}$ (moment 3 in figures 5*b* and 6*b*). Therefore, the Δ*V*/*V*_0_-*t*/*τ*_r_ curve (figure 10*a*) shows no delay time after the shell is released. Correspondingly, the $\Delta P_{\text{min}}/E_{0}$ decreases to zero at *t*_creep_/*τ*_r_ = 0.6134 and starts to increase, indicating that the shell stays monostable during the holding process. The shells for both *t*_creep_/*τ*_r_ = 1 and 5 exhibit pseudo-bistable behaviour (figure 10*a*), and the shell for *t*_creep_/*τ*_r_ = 5 shows a longer recovery time *t*_rec_ than the one for *t*_creep_/*τ*_r_ = 1. This is because a longer time of holding process results in a smaller $\Delta P_{\text{min}}/E_{0}$, and therefore a longer time is needed for $\Delta P_{\text{min}}/E_{0}$ to recover positive (figure 10*b*). In addition, figure 10*b* shows that $\Delta P_{\text{min}}/E_{0}$ decreases more and more slowly as the time of holding process increases and almost reaches a plateau when *t*_creep_/*τ*_r_ = 5. This indicates that *t*_rec_ also approaches a plateau as *t*_creep_/*τ*_r_ becomes very long.

**Figure 11.**
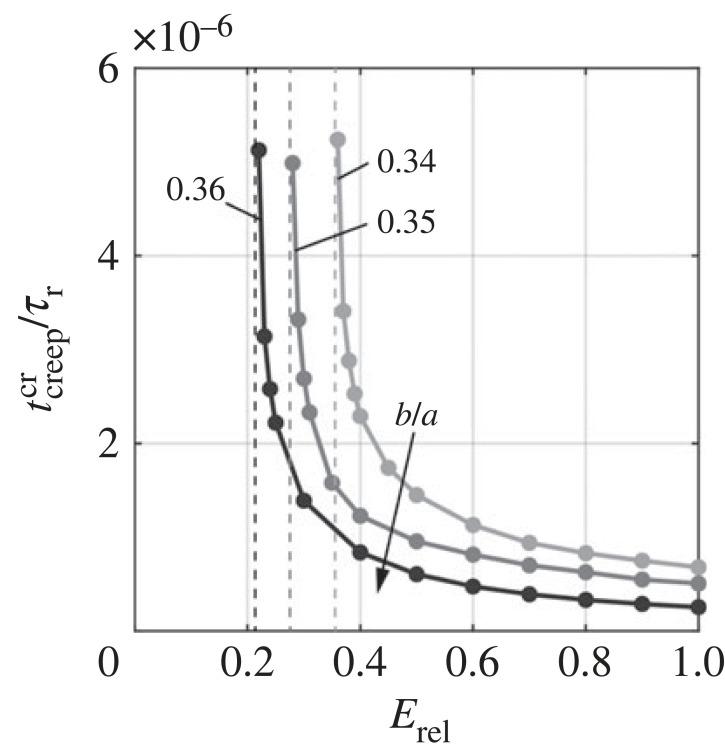
Dependence of the critical creeping time $t_{\text{creep}}^{\text{cr}}$ on relative modulus of relaxation *E*_rel_ and minor-to-major-length ratios *b*/*a*. The dashed lines represent the critical values of *E*_rel_ for $t_{\text{creep}}^{\text{cr}}$ to asymptotically reach infinity.

**Figure 12.**
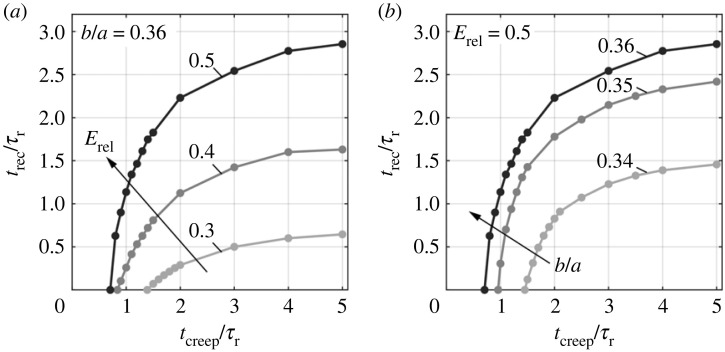
Dependence of the recovery time *t*_rec_ on the creep time *t*_creep_ for different (*a*) relative modulus of relaxation *E*_rel_ and (*b*) minor-to-major-length ratios *b*/*a*.

We summarize the effect of geometry, viscoelastic property and loading history on the pseudo-bistable behaviour in figures 11 and 12. In figure 11, we show the effect of minor-to-major-length ratio *b*/*a* and relative modulus of relaxation *E*_rel_ on the critical creeping time $t_{\text{creep}}^{\text{cr}}$. When *b*/*a* = 0.36, $t_{\text{creep}}^{\text{cr}}$ increases with the decrease of *E*_rel_, and goes to infinity as *E*_rel_ approaches 0.214 (figure 11), indicating an infinite time of holding required for pseudo-bistability. The viscoelastic shells with *E*_rel_ below 0.214 can never exhibit pseudo-bistability no matter how long the pressure is held, since the viscoelastic effect is not strong enough. As *b*/*a* decreases, the corresponding asymptotic line (dashed line in figure 11) is shifted to the right, meaning that a shallow shell (lower *b*/*a*) needs a stronger viscoelastic effect (larger *E*_rel_) for the stability transition to occur. For a given *E*_rel_, a deeper shell has a shorter $t_{\text{creep}}^{\text{cr}}$, and thus requires a shorter holding time to acquire pseudo-bistability. Figure 12 illustrates the influence of *t*_creep_/*τ_r_*, *b*/*a* and *E*_rel_ on the recovery time *t*_rec_/*τ_r_*. We find *t*_rec_/*τ_r_* increases with *t*_creep_/*τ_r_* and saturates when *t*_creep_/*τ_r_* becomes much longer than 1, regardless of *b*/*a* and *E*_rel_. For fixed *b*/*a* = 0.36, a higher *E*_rel_ results in a longer *t*_rec_/*τ_r_*, and requires a shorter *t*_creep_/*τ_r_* to trigger the pseudo-bistable behaviour (*t*_rec_/*τ_r_* > 0) (figure 12*a*). Moreover, *b*/*a* also has a strong influence on *t*_rec_/*τ_r_* (figure 12*b*). We find that a deeper monostable shell with higher *b*/*a*, which is closer to that of bistable shells, leads to a more significant delay time with longer *t*_rec_/*τ_r_*. The effects of *t*_creep_/*τ_r_*, *b*/*a* and *E*_rel_ on *t*_rec_/*τ_r_* mentioned above are consistent with the FEA simulations and experimental observations reported in literature [16,18,21,22].

## 6. Conclusion

In this paper, we model and explain the pseudo-bistable behaviour of viscoelastic shells with a viscoelastic shell model. The model combines the small strain, moderate rotation shell theory with the standard linear solid as the viscoelastic constitutive law. The equilibrium equations are derived by using the principle of virtual work based on the assumption of axisymmetric deformation. By numerically solving the equilibrium equations, the time-dependent buckling behaviors of viscoelastic shells far beyond the buckling point are obtained.

As an example, we apply the proposed model to investigate viscoelastic ellipsoidal shells. Time-dependent buckling analyses are conducted for them under volume-controlled loading conditions. The viscoelastic shells loaded extremely fast (slow) exhibit pressure-volume change relations approaching those of the elastic shells with the short-time elastic modulus *E*_1_ + *E*_∞_ (long-time elastic modulus *E*_∞_). For a moderate loading rate, the pressure-volume change curve shifts downward as either the loading rate decreases or the relative relaxation modulus *E*_rel_ increases. Correspondingly, the critical pressure for buckling decreases.

Using the proposed viscoelastic shell model, we successfully predict the pseudo-bistable behaviour and reveal its mechanism by quantitatively probing the stability transition of viscoelastic shells during a process of holding and releasing a pressure. We first apply an instantaneous pressure sufficient to buckle a monostable shell, hold the pressure for a certain amount of time, and then remove it. With an appropriate choice of shallowness and viscoelasticity, the buckled shell creeps while the pressure is held, stays inverted after the pressure is removed, and finally recovers from its inverted state after a delay time. The characteristics of this time-dependent deformation agree with those obtained from FEA and experiments in literature. Moreover, the viscoelastic shell model allows us to produce the evolution of the instantaneous pressure-volume change relation, which indicates the stability of the shell, at different times during the holding and releasing process. We observe that the shell's stability transitions from a monostable state, temporarily bistable state and eventually back to the monostable state. This observation confirms the mechanism of the pseudo-bistability phenomenon. Finally, we conduct a parametric study to investigate the influence of geometry, viscoelastic property and loading history on the pseudo-bistable behaviour. We find that a shallower shell requires a longer time of holding to achieve pseudo-bistability, and that the recovery time can be increased by either enlarging the viscoelastic relaxation or reducing the shallowness closer to that of bistable shells.

## Data Availability

The codes and the supplementary video are provided in electronic supplementary material [29].

## Appendix A. Derivation of equilibrium equations using the principle of virtual work

In this section, the derivation for the equilibrium equations in equation (2.11) is presented in detail. The internal virtual work (IVW) can be expressed as,

A 1 $$\text{IVW}=\int_{S}\left[N^{\alpha \beta }\delta E_{\alpha \beta }+M^{\alpha \beta }\delta K_{\alpha \beta }\right]\;\text{d}S,$$

where *δE_αβ_* and *δK_αβ_* are the virtual strain components, *S* denotes the area of the mid-surface of the shell. Based on the small strain, moderate rotation shell theory [26,27], *δE_αβ_* and *δK_αβ_* can be written as

A 2 $$\begin{array}{l} \;\delta E_{\alpha \beta }=\frac{1}{2}(\delta u_{\alpha ,\beta }+\delta u_{\beta ,\alpha })+b_{\alpha \beta }\delta w+\varphi_{\alpha }\delta \varphi_{\beta }+g_{\alpha \beta }\phi \delta \phi \\ \;\text{and}\;\delta K_{\alpha \beta }=-\delta w_{,\alpha \beta }+b_{\alpha \gamma ,\beta }\delta u^{\gamma }-\frac{1}{4}(b_{\beta }^{\gamma }\delta u_{\alpha ,\gamma }+b_{\alpha }^{\gamma }\delta u_{\beta ,\gamma })+\frac{3}{4}(b_{\beta \gamma }\delta u_{,\alpha }^{\gamma }+b_{\alpha \gamma }\delta u_{,\beta }^{\gamma }), \end{array}\}$$

where

A 3 $$\begin{array}{ll} & \varphi_{\alpha }=-w_{,\alpha }+b_{\alpha }^{\gamma }u_{\gamma },\delta \varphi_{\alpha }=-\delta w_{,\alpha }+b_{\alpha }^{\gamma }\delta u_{\gamma },\phi =\frac{1}{2}\epsilon^{\alpha \beta }u_{\beta ,\alpha },\delta \phi =\frac{1}{2}\epsilon^{\alpha \beta }\delta u_{\beta ,\alpha },\\ & \epsilon^{\alpha \beta }=\{\begin{array}{lll} 1/\sqrt{g}, & \text{when} & \alpha =1,\beta =2\\ 0, & \text{when} & \alpha =\beta \\ -1/\sqrt{g}, & \text{when} & \alpha =2,\beta =1 \end{array},\;g=|g_{\alpha \beta }|. \end{array}$$

Substituting equation (A 2) into equation (A 1), the internal virtual work can be rewritten as

A 4 $$\begin{array}{rl} \text{IVW} & \;=\int_{S}\{[-M_{,\alpha \beta }^{\alpha \beta }+N^{\alpha \beta }b_{\alpha \beta }+{\left(N^{\alpha \beta }\varphi_{\alpha }\right)}_{,\beta }]\delta w+[-N_{,\beta }^{\gamma \beta }-M_{,\beta }^{\alpha \beta }b_{\alpha }^{\gamma }-\frac{1}{2}{\left(M^{\alpha \beta }b_{\alpha }^{\gamma }-M^{\gamma \alpha }b_{\alpha }^{\beta }\right)}_{,\beta }\\ & \;+N^{\alpha \beta }\varphi_{\alpha }b_{\beta }^{\gamma }-\frac{1}{2}{\left(N_{\alpha }^{\alpha }\phi \epsilon^{\mu \gamma }\right)}_{,\mu }]\delta u_{\gamma }\}\text{d}S\\ & \;\;+\oint_{C}\{-M^{\alpha \beta }n_{\beta }n_{\alpha }\delta w_{,n}+[M_{,\beta }^{\alpha \beta }n_{\alpha }+{\left(M^{\alpha \beta }n_{\beta }t_{\alpha }\right)}_{,t}-N^{\alpha \beta }\varphi_{\alpha }n_{\beta }]\delta w\\ & \;+\left(N^{\gamma \beta }n_{\beta }+\frac{3}{2}M^{\alpha \beta }n_{\beta }b_{\alpha }^{\gamma }-\frac{1}{2}M^{\gamma \beta }b_{\beta }^{\alpha }n_{\alpha }+\frac{1}{2}N_{\alpha }^{\alpha }\phi \epsilon^{\mu \gamma }n_{\mu }\right)\delta u_{\gamma }\}\text{d}s-M^{\alpha \beta }n_{\beta }t_{\alpha }\delta w{|}_{\text{corners}}, \end{array}$$

where ()_,_*_α_* and ()_,_*_αβ_* are the first- and second-order covariant derivatives of (), *C* is the boundary of the mid-surface, *n_α_* and *t_α_* represent the components of the unit vectors normal and tangent to the edge *C*, respectively. The external virtual work (EVW) due to a uniform pressure acting on the shell in the deformed state (called live pressure) is [27]

A 5 $$\text{EVW}=\int_{\bar{S}}\Delta P\bar{N}\cdot (\delta u^{\beta }x_{,\beta }+\delta wN)\;\text{d}\bar{S}+\oint_{C}\left(T^{\gamma }\delta u_{\gamma }+Q\delta w-M_{n}\delta w_{,n}\right)\;\text{d}s,$$

where $\bar{N}$ denotes the unit vector normal to the mid-surface of the deformed shell, $\bar{S}$ denotes the area of the mid-surface of the shell in the deformed state. Under the condition of small strain and moderate rotation, the EVW becomes

A 6 $$\begin{array}{rl} \text{EVW} & \;=\int_{S}\left[\Delta P(1+u_{,\gamma }^{\gamma }+b_{\gamma }^{\gamma }w)\delta w+\Delta P(\varphi^{\gamma }+\phi \varphi^{\eta }\epsilon_{\eta }^{\gamma })\delta u_{\gamma }\right]\;\text{d}S+\oint_{C}(T^{\gamma }\delta u_{\gamma }+Q\delta w-M_{n}\delta w_{,n})\;\text{d}s. \end{array}$$

By enforcing IVW = EVW, we can obtain the following equilibrium equations

A 7 $$\begin{array}{rl} & \;-M_{,\alpha \beta }^{\alpha \beta }+N^{\alpha \beta }b_{\alpha \beta }+{\left(N^{\alpha \beta }\varphi_{\alpha }\right)}_{,\beta }=\Delta P(1+u_{,\gamma }^{\gamma }+b_{\gamma }^{\gamma }w),\\ & \;\;-N_{,\beta }^{\gamma \beta }-M_{,\beta }^{\alpha \beta }b_{\alpha }^{\gamma }-\frac{1}{2}{\left(M^{\alpha \beta }b_{\alpha }^{\gamma }-M^{\gamma \alpha }b_{\alpha }^{\beta }\right)}_{,\beta }+N^{\alpha \beta }\varphi_{\alpha }b_{\beta }^{\gamma }-\frac{1}{2}{\left(N_{\alpha }^{\alpha }\phi \epsilon^{\mu \gamma }\right)}_{,\mu }\\ & \;\;=\Delta P(\varphi^{\gamma }+\phi \varphi^{\eta }\epsilon_{\eta }^{\gamma }), \end{array}$$

and boundary conditions:

A 8 $$\begin{array}{rl} & \text{Specify}\;N^{\gamma \beta }n_{\beta }+\frac{3}{2}M^{\alpha \beta }n_{\beta }b_{\alpha }^{\gamma }-\frac{1}{2}M^{\gamma \beta }b_{\beta }^{\alpha }n_{\alpha }+\frac{1}{2}N_{\alpha }^{\alpha }\phi \epsilon^{\mu \gamma }n_{\mu }=T^{\gamma }\;\text{or}\;u_{\gamma }\\ & \text{Specify}\;M^{\alpha \beta }n_{\beta }n_{\alpha }=M_{n}\;\text{or}\;w_{,n}\\ & \text{Specify}\;M_{,\beta }^{\alpha \beta }n_{\alpha }+{\left(M^{\alpha \beta }n_{\beta }t_{\alpha }\right)}_{,t}-N^{\alpha \beta }\varphi_{\alpha }n_{\beta }=Q\;\text{or}\;w. \end{array}$$

The term − *M^αβ^n_β_t_α_δw*|_corners_ in equation (A 4) is related to the virtual work of concentrated loads at any corners.

Since we only consider axisymmetric deformation, *u^θ^* and *w* are only functions of *θ*, *u^ω^* = 0, *φ**^ω^* = *φ**_ω_* = 0, *ϕ* = 0. With the assumption of axisymmetric deformation, each term in equation (A 7) can be written as

A 9 $$\begin{array}{rl} -M_{,\alpha \beta }^{\alpha \beta } & \;=M^{\omega \omega }(-{\Gamma_{\omega \omega }^{\theta }}^{\prime }-\Gamma_{\theta \theta }^{\theta }\Gamma_{\omega \omega }^{\theta }-\Gamma_{\omega \omega }^{\theta }\Gamma_{\theta \omega }^{\omega })\\ & \;\;+M^{\theta \theta }[-{\left(\Gamma_{\theta \omega }^{\omega }\right)}^{2}-2{\Gamma_{\theta \theta }^{\theta }}^{\prime }-2{\left(\Gamma_{\theta \theta }^{\theta }\right)}^{2}-{\Gamma_{\theta \omega }^{\omega }}^{\prime }-3\Gamma_{\theta \theta }^{\theta }\Gamma_{\theta \omega }^{\omega }]\\ & \;\;-{M^{\omega \omega }}^{\prime }\Gamma_{\omega \omega }^{\theta }+{M^{\theta \theta }}^{\prime }(-3\Gamma_{\theta \theta }^{\theta }-2\Gamma_{\theta \omega }^{\omega })-{M^{\theta \theta }}^{\prime \prime }, \end{array}$$

A 10 $$\begin{array}{r} N^{\alpha \beta }b_{\alpha \beta }=N^{\omega \omega }b_{\omega }^{\omega }g_{\omega \omega }+N^{\theta \theta }b_{\theta }^{\theta }g_{\theta \theta }, \end{array}$$

A 11 $$\begin{array}{rl} {\left(N^{\alpha \beta }\varphi_{\alpha }\right)}_{,\beta } & \;=-N^{\omega \omega }\varphi g_{\theta \theta }\Gamma_{\omega \omega }^{\theta }+({N^{\theta \theta }}^{\prime }+N^{\theta \theta }\Gamma_{\theta \theta }^{\theta })\varphi g_{\theta \theta }+N^{\theta \theta }(\varphi^{\prime }g_{\theta \theta }+\varphi {g_{\theta \theta }}^{\prime })\\ & \;\;+\varphi g_{\theta \theta }(N^{\omega \omega }\Gamma_{\omega \omega }^{\theta }+N^{\theta \theta }\Gamma_{\theta \omega }^{\omega }), \end{array}$$

A 12 $$\begin{array}{rl} & \Delta P(1+u_{,\gamma }^{\gamma }+b_{\gamma }^{\gamma }w)=\Delta P[1+u^{\theta }\Gamma_{\theta \omega }^{\omega }+u^{\theta^{\prime }}+u^{\theta }\Gamma_{\theta \theta }^{\theta }+(b_{\omega }^{\omega }+b_{\theta }^{\theta })w], \end{array}$$

A 13 $$\begin{array}{rl} & \;-N_{,\beta }^{\gamma \beta }=-N_{,\beta }^{\theta \beta }=-N^{\omega \omega }\Gamma_{\omega \omega }^{\theta }-N^{\theta \theta }\Gamma_{\theta \omega }^{\omega }-{N^{\theta \theta }}^{\prime }-2N^{\theta \theta }\Gamma_{\theta \theta }^{\theta }, \end{array}$$

A 14 $$\begin{array}{rl} & \;-M_{,\beta }^{\alpha \beta }b_{\alpha }^{\gamma }=-M_{,\beta }^{\alpha \beta }b_{\alpha }^{\theta }=-b_{\theta }^{\theta }(M^{\omega \omega }\Gamma_{\omega \omega }^{\theta }+M^{\theta \theta }\Gamma_{\theta \omega }^{\omega }+{M^{\theta \theta }}^{\prime }+2M^{\theta \theta }\Gamma_{\theta \theta }^{\theta }), \end{array}$$

A 15 $$\begin{array}{rl} & \;-\frac{1}{2}{\left(M^{\alpha \beta }b_{\alpha }^{\gamma }-M^{\gamma \alpha }b_{\alpha }^{\beta }\right)}_{,\beta }=-\frac{1}{2}{\left(M^{\alpha \beta }b_{\alpha }^{\theta }-M^{\theta \alpha }b_{\alpha }^{\beta }\right)}_{,\beta }=0, \end{array}$$

A 16 $$\begin{array}{rl} & N^{\alpha \beta }\varphi_{\alpha }b_{\beta }^{\gamma }=N^{\alpha \beta }\varphi_{\alpha }b_{\beta }^{\theta }=N^{\theta \theta }b_{\theta }^{\theta }\varphi g_{\theta \theta }, \end{array}$$

A 17 $$\begin{array}{rl} & \;-\frac{1}{2}{\left(N_{\alpha }^{\alpha }\phi \epsilon^{\mu \gamma }\right)}_{,\mu }=-\frac{1}{2}{\left(N_{\alpha }^{\alpha }\phi \epsilon^{\mu \theta }\right)}_{,\mu }=0 \end{array}$$

A 18 $$\begin{array}{r} \;\text{and}\;\Delta P(\varphi^{\gamma }+\phi \varphi^{\eta }\epsilon_{\eta }^{\gamma })=\Delta P(\varphi^{\theta }+\phi \varphi^{\eta }\varepsilon_{\eta }^{\theta })=\Delta P\varphi , \end{array}$$

where (·)′ denotes d(·)/d*θ*, *φ = φ^θ^*, *g_αβ_* and *g^αβ^* are the covariant and contravariant components of the first fundamental form of the mid-surface,

A 19 $$\begin{array}{rl} g_{\omega \omega } & \;=\frac{1}{g^{\omega \omega }}=x_{,\omega }\cdot x_{,\omega }=R^{2}\cos^{2}\theta ,\;g_{\theta \theta }=\frac{1}{g^{\theta \theta }}=x_{,\theta }\cdot x_{,\theta }={R^{\prime }}^{2}+R^{2}.\\ g_{\omega \theta } & \;=g_{\theta \omega }=g^{\omega \theta }=g^{\theta \omega }=0, \end{array}$$

*b_αβ_* and $b_{\beta }^{\alpha }$ are the covariant and the mixed components of the second fundamental form of the mid-surface,

A 20 $$\begin{array}{rl} b_{\omega \omega } & \;=-N\cdot x_{,\omega \omega }=\frac{R\cos\theta (R^{\prime }\sin\theta +R\cos\theta )}{\sqrt{{R^{\prime }}^{2}+R^{2}}},\;b_{\omega }^{\omega }=b_{\omega \omega }g^{\omega \omega }=\frac{R^{\prime }\sin\theta +R\cos\theta }{R\cos\theta \sqrt{{R^{\prime }}^{2}+R^{2}}}\\ b_{\theta \theta } & \;=-N\cdot x_{,\theta \theta }=\frac{2{R^{\prime }}^{2}-RR^{\prime \prime }+R^{2}}{\sqrt{{R^{\prime }}^{2}+R^{2}}},\;b_{\theta }^{\theta }=b_{\theta \theta }g^{\theta \theta }=\frac{\left(2{R^{\prime }}^{2}-RR^{\prime \prime }+R^{2}\right)}{{\left({R^{\prime }}^{2}+R^{2}\right)}^{\left(3/2\right)}},\\ b_{\omega \theta } & \;=b_{\theta \omega }=b_{\theta }^{\omega }=b_{\omega }^{\theta }=0, \end{array}$$

and $\Gamma_{\alpha \beta }^{\gamma }$ denotes the Christoffel symbols,

A 21 $$\Gamma_{\alpha \beta }^{\gamma }=\frac{1}{2}g^{\gamma \lambda }\left(\frac{\partial g_{\alpha \lambda }}{\partial \beta }+\frac{\partial g_{\beta \lambda }}{\partial \alpha }-\frac{\partial g_{\alpha \beta }}{\partial \lambda }\right)$$

yielding the following non-zero components

A 22 $$\Gamma_{\theta \omega }^{\omega }=\Gamma_{\omega \theta }^{\omega }=\frac{R^{\prime }\cos\theta -R\sin\theta }{R\cos\theta },\;\Gamma_{\theta \theta }^{\theta }=\frac{R^{\prime }R^{\prime \prime }+RR^{\prime }}{{R^{\prime }}^{2}+R^{2}},\;\Gamma_{\omega \omega }^{\theta }=\frac{-RR^{\prime }\cos^{2}\theta +R^{2}\cos\theta \sin\theta }{{R^{\prime }}^{2}+R^{2}}.$$

## References

[1] Hutchinson JW. 2016 Buckling of spherical shells revisited. Proc. R. Soc. Math. Phys. Eng. Sci. **472**, 20160577. (10.1098/rspa.2016.0577)

[2] Ramm E, Wall W. 2004 Shell structures—a sensitive interrelation between physics and numerics. Int. J. Numer. Methods Eng. **60**, 381-427. (10.1002/nme.967)

[3] Bushnell D. 1981 Buckling of shells-pitfall for designers. AIAA J. **19**, 1183-1226. (10.2514/3.60058)

[4] Nemeth MP, Starnes Jr JH. 1998 The NASA monographs on shell stability design recommendations: a review and suggested improvements.

[5] Katifori E, Alben S, Cerda E, Nelson DR, Dumais J. 2010 Foldable structures and the natural design of pollen grains. Proc. Natl Acad. Sci. USA **107**, 7635-7639. (10.1073/pnas.0911223107) 20404200 PMC2867878

[6] Forterre Y, Skotheim JM, Dumais J, Mahadevan L. 2005 How the Venus flytrap snaps. Nature **433**, 421-425. (10.1038/nature03185) 15674293

[7] Reis PM. 2015 A perspective on the revival of structural (in) stability with novel opportunities for function: from buckliphobia to buckliphilia. J. Appl. Mech. **82**, 111001. (10.1115/1.4031456)

[8] Bertoldi K, Vitelli V, Christensen J, van Hecke M. 2017 Flexible mechanical metamaterials. Nat. Rev. Mater. **2**, 17066. (10.1038/natrevmats.2017.66)

[9] Qiao C, Liu L, Pasini D. 2021 Bi-shell valve for fast actuation of soft pneumatic actuators via shell snapping interaction. Adv. Sci. **8**, 2100445. (10.1002/advs.202100445) PMC8336518 34061464

[10] Gorissen B, Melancon D, Vasios N, Torbati M, Bertoldi K. 2020 Inflatable soft jumper inspired by shell snapping. Sci. Robot. **5**, eabb1967. (10.1126/scirobotics.abb1967) 33022625

[11] Bartlett NW, Tolley MT, Overvelde JT, Weaver JC, Mosadegh B, Bertoldi K, Whitesides GM, Wood RJ. 2015 A 3D-printed, functionally graded soft robot powered by combustion. Science **349**, 161-165. (10.1126/science.aab0129) 26160940

[12] Chi Y, Hong Y, Zhao Y, Li Y, Yin J. 2022 Snapping for high-speed and high-efficient butterfly stroke–like soft swimmer. Sci. Adv. **8**, eadd3788. (10.1126/sciadv.add3788) 36399554 PMC9674291

[13] Preston DJ, Rothemund P, Jiang HJ, Nemitz MP, Rawson J, Suo Z, Whitesides GM. 2019 Digital logic for soft devices. Proc. Natl Acad. Sci. USA **116**, 7750-7759. (10.1073/pnas.1820672116) 30923120 PMC6475414

[14] Rothemund P, Ainla A, Belding L, Preston DJ, Kurihara S, Suo Z, Whitesides GM. 2018 A soft, bistable valve for autonomous control of soft actuators. Sci. Robot. **3**, eaar7986. 33141749 10.1126/scirobotics.aar7986

[15] Holmes DP, Crosby AJ. 2007 Snapping surfaces. Adv. Mater. **19**, 3589-3593. (10.1002/adma.200700584)

[16] Brinkmeyer A, Santer M, Pirrera A, Weaver PM. 2012 Pseudo-bistable self-actuated domes for morphing applications. Int. J. Solids Struct. **49**, 1077-1087. (10.1016/j.ijsolstr.2012.01.007)

[17] Gomez M, Moulton DE, Vella D. 2019 Dynamics of viscoelastic snap-through. J. Mech. Phys. Solids **124**, 781-813. (10.1016/j.jmps.2018.11.020)

[18] Santer M. 2010 Self-actuated snap back of viscoelastic pulsing structures. Int. J. Solids Struct. **47**, 3263-3271. (10.1016/j.ijsolstr.2010.08.007)

[19] Brinkmeyer A, Pirrera A, Santer M, Weaver P. 2013 Pseudo-bistable pre-stressed morphing composite panels. Int. J. Solids Struct. **50**, 1033-1043. (10.1016/j.ijsolstr.2012.11.019)

[20] Urbach EY, Efrati E. 2020 Predicting delayed instabilities in viscoelastic solids. Sci. Adv. **6**, eabb2948. (10.1126/sciadv.abb2948) 32917615 PMC7473665

[21] Liu T, Chen Y, Liu L, Liu Y, Leng J, Jin L. 2021 Effect of imperfections on pseudo-bistability of viscoelastic domes. Extreme Mech. Lett. **49**, 101477. (10.1016/j.eml.2021.101477)

[22] Chen Y, Liu T, Jin L. 2022 Spatiotemporally programmable surfaces via viscoelastic shell snapping. Adv. Intell. Syst. **4**, 2100270. (10.1002/aisy.202100270)

[23] Che K, Rouleau M, Meaud J. 2019 Temperature-tunable time-dependent snapping of viscoelastic metastructures with snap-through instabilities. Extreme Mech. Lett. **32**, 100528. (10.1016/j.eml.2019.100528)

[24] Dykstra DM, Janbaz S, Coulais C. 2022 The extreme mechanics of viscoelastic metamaterials. APL Mater. **10**, 080702. (10.1063/5.0094224)

[25] Liu T, Chen Y, Hutchinson JW, Jin L. 2022 Buckling of viscoelastic spherical shells. J. Mech. Phys. Solids **169**, 105084. (10.1016/j.jmps.2022.105084)

[26] Sanders Jr JL. 1963 Nonlinear theories for thin shells. Q. Appl. Math. **21**, 21-36. (10.1090/qam/147023)

[27] Budiansky B. 1968 Notes on nonlinear shell theory. J. Appl. Mech. **35**, 393-401. (10.1115/1.3601208)

[28] Koiter W. 1969 Nonlinear buckling problem of a complete spherical shell under uniform external pressure. I. Proc. K. Ned. Akad. Van Wet. Ser. B-Phys. Sci. **72**, 40.

[29] Chen Y, Liu T, Jin L. 2023 Pseudo-bistability of viscoelastic shells. Figshare. (10.6084/m9.figshare.c.6404420) PMC9922547 36774958

